# Oncolytic Virotherapy for Melanoma Brain Metastases, a Potential New Treatment Paradigm?

**DOI:** 10.3390/brainsci11101260

**Published:** 2021-09-23

**Authors:** Sauson Soldozy, Kathleen M. Mulligan, David X. Zheng, Melissa A. Levoska, Christopher R. Cullison, Turki Elarjani, Daniel G. Eichberg, Leonel E. Ampie, Ashish H. Shah, Kaan Yağmurlu, Mark E. Shaffrey, Jeffrey F. Scott, Ricardo J. Komotar

**Affiliations:** 1 Department of Neurological Surgery, University of Virginia Health System, Charlottesville, VA 22903, USA; ss2ah@virginia.edu (S.S.); leonel.ampie@nih.gov (L.E.A.); kaan_yagmur@yahoo.com (K.Y.); mes8c@hscmail.mcc.virginia.edu (M.E.S.); 2 Department of Neurological Surgery, University of Miami, Miami, FL 33146, USA; telarjani@gmail.com (T.E.); daniel.eichberg@jhsmiami.org (D.G.E.); AShah@med.miami.edu (A.H.S.); 3 Department of Dermatology, Case Western Reserve University School of Medicine, Cleveland, OH 44106, USA; kmm334@case.edu (K.M.M.); dxz281@case.edu (D.X.Z.); melissa.levoska@gmail.com (M.A.L.); cxc886@case.edu (C.R.C.); 4 Surgical Neurology Branch, National Institute of Neurological Disorders and Stroke, National Institutes of Health, Bethesda, MD 20824, USA; 5 Department of Dermatology, Johns Hopkins University School of Medicine, Baltimore, MD 21205, USA; jscott98@jhmi.edu

**Keywords:** oncolytic virotherapy, melanoma, brain metastases, neuroimmunology, neuro-oncology

## Abstract

Introduction: Melanoma brain metastases remain a devastating disease process with poor prognosis. Recently, there has been a surge in studies demonstrating the efficacy of oncolytic virotherapy for brain tumor treatment. Given their specificity and amenability to genetic modification, the authors explore the possible role of oncolytic virotherapy as a potential treatment option for patients with melanoma brain metastases. Methods: A comprehensive literature review including both preclinical and clinical evidence of oncolytic virotherapy for the treatment of melanoma brain metastasis was performed. Results: Oncolytic virotherapy, specifically T-VEC (Imlygic™), was approved for the treatment of melanoma in 2015. Recent clinical trials demonstrate promising anti-tumor changes in patients who have received T-VEC; however, there is little evidence for its use in metastatic brain disease based on the existing literature. To date, only two single cases utilizing virotherapy in patients with metastatic brain melanoma have been reported, specifically in patients with treatment refractory disease. Currently, there is not sufficient data to support the use of T-VEC or other viruses for intracranial metastatic melanoma. In developing a virotherapy treatment paradigm for melanoma brain metastases, several factors must be considered, including route of administration, need to bypass the blood–brain barrier, viral tumor infectivity, and risk of adverse events. Conclusions: Evidence for oncolytic virotherapy treatment of melanoma is limited primarily to T-VEC, with a noticeable paucity of data in the literature with respect to brain tumor metastasis. Given the promising findings of virotherapy for other brain tumor types, oncolytic virotherapy has great potential to offer benefits to patients afflicted with melanoma brain metastases and warrants further investigation.

## 1. Introduction

Melanoma, a cancer typically arising from melanocytes in the basal layer of the epidermis, is a common source of metastatic disease to the central nervous system (CNS) [1]. Brain metastases may occur in 10–40% of patients with melanoma depending on the stage at diagnosis, rising to 73% in autopsy series [2,3]. Diagnosis may be further complicated by patients having metastatic disease without a known primary lesion, occurring in roughly 5% of patients [4]. Between 40–60% of melanoma tumors possess a mutation in the *BRAF* gene, resulting in oncogenic proliferation mediated by activation of the mitogen-activated protein kinase (MAPK) pathway [5,6]. Up to 95% of *BRAF*-mutant melanomas have the V600E mutation, which confers an approximately 12% increased risk of brain metastases compared to wildtype patients [7,8].

A greater understanding of melanoma oncoprogression has led to the introduction of several new interventions within the last decade. These include targeted therapies such as vemurafenib and dabrafenib, both *BRAF* inhibitors with demonstrated clinical efficacy in treating brain metastases carrying the *BRAF* V600E mutation [9]. Immunotherapies including high-dose interleukin-2 (IL-2) and immune checkpoint inhibitors that target programmed death 1 (PD-1) and its ligand (PD-L1) (e.g., nivolumab, pembrolizumab) have also been introduced [10,11,12,13,14,15,16].

Despite these new therapeutics, prognosis remains poor in patients with metastatic brain melanoma with a median overall survival of 4.4 to 4.7 months from time of brain metastasis diagnosis [3], ranging from 5 to 9 months in patients receiving *BRAF* inhibitors [17,18,19]. Combined surgery/radiotherapy in conjunction with targeted therapy or immunotherapy portends survival beyond 12 months, in some cases. However, resistance to *BRAF* inhibitors often develops [20]. Moreover, intracranial hemorrhage, among other systemic comorbidities, has been reported with these agents [1]. Relative to the body of evidence surrounding the clinical efficacy of *BRAF* inhibitors, evidence regarding immunomodulating antibodies is limited secondary to exclusion from clinical trials, with reports of inflammatory reactions within the brain [11,12,13,14,15,16]. Additionally, the necessity of corticosteroids to control cerebral edema in some patients raises concern for the reduced efficacy of immune checkpoint antibodies due to an attenuated immune response from steroids.

**Figure 1:**
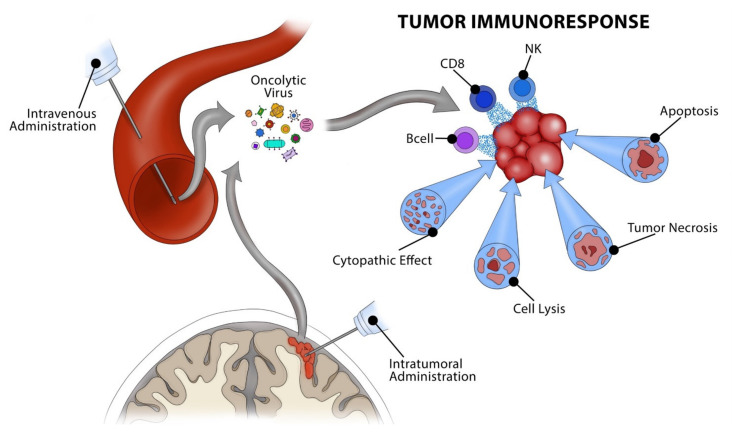
Depiction of oncolytic virotherapy for treatment of brain tumors. Viruses can be administered either systemically through intravenous injection or directly into the tumor or tumor bed status-post resection. Oncolytic viruses preferentially target and selectively infect tumor cells, triggering tumor cell death through a variety of mechanisms including induction of apoptosis, direct cell lysis, or through recruitment of local immune mediators. Artwork courtesy of Roberto C. Suazo, Medical Illustrator and Graphic Design Project Manager for the Department of Neurological Surgery, University of Miami.

Patients with brain metastasis are often excluded from major melanoma clinical trials secondary to concerns of overall poor survival, and, arguably the largest barrier to systemic treatment, concerns related to blood–brain barrier (BBB) drug penetration [21]. This contributes to a lack of data, and therefore treatments for melanoma brain metastasis are extrapolated mainly based on results of clinical trials involving only extracranial metastases [21]. This is especially problematic, given evidence that the tumor microenvironment of the CNS induces molecular and genetic changes in melanoma cells, contributing to increased resistance and ineffectiveness of targeted therapies against brain metastases [22,23,24,25,26]. Thus, there is a need to explore alternative treatment options with demonstrated sufficient CNS penetrance and tolerability that do not overburden patients with systemic cytotoxicity. Oncolytic virotherapy (OV), a newly emerging form of immunotherapy, may serve to fill this niche and warrants further exploration, especially given evidence that certain viruses can effectively cross the BBB (Figure 1) [27,28,29,30,31,32,33,34,35,36,37].

OV has shown great promise in treating CNS malignancies, including high-grade glioma (HGG) [38,39,40,41,42,43]. Many viral strains are available for genetically modified use to selectively infect cancer cells, which can be administered either locally within the tumor site or systemically to stimulate an anti-tumor immune response [44]. In this review, we survey the literature for reports of OV, either preclinical or clinical, to explore its potential role in treating metastatic melanoma disease to the brain.

## 2. Oncolytic Virotherapy

The oncolytic properties of viruses were discovered based on chance observation in the early 1900s with a more formal investigation of these viruses in the late 20th century, following the advent of recombinant DNA technology [38,45,46]. OVs are advantageous in that they are amenable to genetic modification with the use of reporter genes enabling specificity and targeting of tumor-specific entry receptors, signaling pathways, and cell surface antigens [47]. OV can be considered to have dual functionality, with one component being the virus’s intrinsic oncolytic properties, and the other being its ability to act as a precise drug delivery tool given its high specificity for genetically programmed molecular targets. Along with melanoma and HGG, other malignancies in which OV has shown promise in clinical trials include pancreatic, bladder, ovarian, prostate, and hepatocellular carcinomas [48].

Herpes simplex virus (HSV) is among the first and most studied oncolytic virotherapeutic hosts [49], and other viruses currently being studied for their potential use in various cancers include poxvirus, reoviruses, and coxsackieviruses [50]. Each virus induces oncolysis through unique mechanisms depending on how they were engineered. While many viruses induce direct lysis of malignant cells, some induce a local inflammatory reaction via enhanced lymphocytic recruitment and infiltration within the tumor microenvironment. Meanwhile, other viruses inhibit cellular migration and invasion, therefore attenuating tumor extravasation and metastasis [51,52,53]. These viruses’ exact activities may vary depending on the exact molecular profile of cancer involved, and characterization of these mechanisms continues to be investigated. In contrast to other therapeutic options, a significant advantage of certain oncolytic viruses is their ability to easily pass the BBB given natural tropisms for neural tissue or ability to utilize immune cells as carriers [27,54,55,56]. This advantage has been frequently demonstrated with HSV, vaccinia virus, reovirus, parvovirus, and adenovirus, among others [49,57,58]. Despite the ability of some oncolytic viruses to cross the BBB, a disadvantage to systemic administration involves patients’ immune systems neutralizing the virus before it reaches the target site in the brain, which would make direct stereotactic injection advantageous in this regard [59]. Our knowledge of OV is continuously expanding. OV represents an exciting new domain for general cancer treatment, and specifically for treatment of melanoma brain metastases.

## 3. Virotherapy and Melanoma

OV is the most recent addition to the arsenal of melanoma treatments, gaining FDA approval in 2015 [42]. It can be used to treat both *BRAF*- and non-*BRAF*-mutated melanomas. While current data are limited on the efficacy of OV for clinical use, specifically in melanoma cases with brain metastases, preliminary studies have begun to emerge in the literature [43,44,45,46].

In 2015, the first oncolytic virotherapeutic agent, talimogene laherparepvec (T-VEC), was approved by the FDA for use in metastatic melanoma [60]. T-VEC is a modified oncolytic HSV that expresses the granulocyte–macrophage colony-stimulating factor (GM-CSF). This virus contains mutations in infectious cell proteins 34.5 and 47, which allow the virus to selectively infect tumor cells and inhibit tumor cell expression of major histocompatibility complex class I antigens. This serves to initiate a specific immune response against only tumor cells infected by the virus [61].

T-VEC can be injected either intralesionally or into local lymph nodes. It is useful in treating non-visceral melanoma metastases, with 34% of non-visceral lesions decreasing over 50% in size [62]. OPTiM, a phase III clinical trial, determined the overall response rates for T-VEC (26.4%) compared to control GM-CSF (5.7%) for patients with melanoma staged up through IVM1a [63]. Since the authors could not find a statistically significant improvement compared to control in patients with lung or other visceral extracranial metastases (i.e., stages IVM1b-IVM1c), the FDA approved T-VEC for use only up to stage IVM1a [64]. It is important to note that the authors reported durable response rates as their outcome measure, which serve as a source of bias given their subjectivity. Another potential source of bias is the high percentage of patients who discontinued treatment at three months, namely 29.2% in the T-VEC cohort and 56.0% in the GM-CSF cohort. Additionally, similarly to clinical trials for other therapeutic agents, stage IVM1d lesions or intracranial metastases were not included. Despite these limitations, the OPTiM trial and subsequent FDA approval of T-VEC represent an encouraging step toward an effective treatment strategy for metastatic brain disease due to melanoma [65].

In addition to T-VEC, non-neurovirulent rhinovirus:poliovirus chimera (PVSRIPO) has been described for treatment-refractory melanoma in a phase I clinical trial showing promising antitumor activity [66]. However, to date, the majority of data regarding virotherapy and melanoma treatment are limited to T-VEC, likely because this is the only FDA-approved oncolytic virus for the treatment of melanoma [67,68,69,70].

## 4. T-VEC Compared to Other Therapies

The emergence of T-VEC as a novel therapy for melanoma is promising, but the question of how it compares to existing therapies is necessary to address. A year after the approval of T-VEC for treating metastatic melanoma, a meta-analysis was performed to compare the overall survival of melanoma patients treated with T-VEC, ipilimumab, or vemurafenib [71]. The study included four randomized controlled trials: OPTiM (T-VEC), MDX0101-20 (ipilimumab), CA184-024 (ipilimumab), and BRIM-3 (vemurafenib) [64,72,73,74]. The authors defined two main cohorts: patients with all stages of the disease and patients without bone, brain, lung, or visceral metastases (i.e., stages IIIb to IVM1a). The study concluded that T-VEC results in superior overall survival compared to ipilimumab and vemurafenib in patients without visceral metastatic disease. This difference is diminished when looking at patients with all stages of the disease. With respect to brain metastases, a small proportion of patients (<10% overall) was included. It is not feasible to extrapolate the effectiveness of T-VEC for patients with metastatic brain disease based on these analyses, as patients with brain metastases were lumped into the “all patients” category. Attempts to isolate and compare only those patients with brain metastases would not be possible due to insufficient statistical power. Additionally, since brain metastases have been shown to behave uniquely compared to extracranial metastases [23], it remains difficult to determine whether T-VEC would also perform similarly to ipilimumab or vemurafenib in these patients.

Several limitations underlying the methodology employed by this meta-analysis deserve mention. Most notably, a traditional network meta-analysis was forgone due to a lack of standard comparators across studies. Instead, two algorithms (modified Korn and two-step Korn methods) were applied to the ipilimumab and vemurafenib studies. For example, the number of patients with visceral metastatic disease varied greatly across studies. Given that patients with visceral disease will have poorer prognosis regardless of treatment choice, the algorithms employed by Quinn et al. attempt to correct overall survival by assigning a calculated modifier based on the following variables: gender, Eastern Cooperative Oncology Group (ECOG) performance status, presence of visceral metastasis, presence of brain metastasis, and lactate dehydrogenase (LDH) status [71]. Fleeman et al. noted a flaw in the algorithmic analysis of Quinn et al. regarding the inclusion of LDH as a coefficient [75].

Given that LDH status is not relevant for patients with non-visceral disease (i.e., stage IIIb to stage IVM1a disease), inclusion of LDH in analysis reduces the size and impact of the other variables, leading to falsely improved relative efficacy of T-VEC. Moreover, the study by Quinn et al. attempts to pool data from both ipilimumab studies, despite vast differences in their treatment arms. Namely, MDX0101-20 was a three-armed trial in which patients received ipilimumab plus glycoprotein 100 (gp100) peptide vaccine, gp100 alone, or ipilimumab alone [72]. In contrast, CA184-024 sought to compare ipilimumab plus dacarbazine versus placebo plus dacarbazine [73]. The meta-analysis by Quinn et al. assumes no treatment effect due to gp100 and dacarbazine and considers patients receiving combination therapy as equal to those receiving ipilimumab only. Finally, differences in dosing and other treatment-related factors contributed to the considerable heterogeneity among these data. The aforementioned assumptions, along with utilization of the Korn model, have been deemed inappropriate by Fleeman et al., who advise caution when interpreting the results from Quinn et al. [75].

Based on these findings, it remains unclear how T-VEC performs when compared to immunotherapeutic agents for patients with intracranial disease, as the current data prevent us from drawing conclusions regarding the efficacy of T-VEC for brain metastases.

## 5. Clinical Efficacy for Intracranial Metastasis

To date, only two case reports have been published demonstrating initial clinical experience utilizing OV. One of the earliest case reports describing utilization of OV in a patient with melanoma brain metastases was published in 2018 and included T-VEC, pembrolizumab, and whole-brain radiotherapy (WBRT) after the initial failure of combined nivolumab/ipilimumab treatment [76]. The patient was a 68-year-old male who presented with multiple extracranial and intracranial metastases, including two right frontal lobe lesions (7.7 mm and 4.3 mm) and a single right cerebellar mass. Despite GammaKnife in conjunction with nivolumab/ipilimumab treatment, the patient developed several new scattered brain metastases. At this time, a roughly 1-month course of WBRT (3750 cGy in 15 daily fractions) was initiated, followed by T-VEC (every two weeks) and pembrolizumab (every three weeks) therapy after completion of the patient’s WBRT course. An inoculation dose of 4 mL of T-VEC was administered in a large axillary mass for a total of 13 treatments over six months. During this time, a brain MRI demonstrated a reduction in metastatic disease burden and attenuated vasogenic edema. The patient reported no significant side effects, with substantially increased tolerability of T-VEC compared to immunotherapy. However, due to systemic extracranial disease progression, T-VEC was discontinued, and roughly six months after discontinuing T-VEC, the patient passed away secondary to complications related to CNS metastases.

A second case report involved utilization of OV in a patient with metastatic brain melanoma of unknown primary [77]. This case was a 60-year-old female patient who presented with severe dizziness triggered by movement and increased fatigue. A brain MRI revealed a contrast-enhancing, well-circumscribed intradural spinal cord lesion at the craniocervical junction. A subtotal resection was achieved. Full-body scans did not show a primary source, and dermatology evaluation did not reveal a cutaneous primary. Despite this, histologic examination was consistent with *BRAF*-negative metastatic melanoma. The patient declined radiotherapy and did not qualify for *BRAF* inhibitors, and immunotherapy was not an option given her residence in Latvia. For this reason, the oncolytic virus Rigivir was pursued as a treatment option. Rigivir, an unmodified Enteric Cytopathogenic Human Orphan type 7 (ECHO-7) picornavirus, was the first OV in the world to gain regulatory approval for the treatment of melanoma, local treatment of skin and subcutaneous melanoma metastases, and prevention of relapse and metastasis after radical surgery [59,78]. However, Rigivir has not yet gained approval in the United States. The patient was initiated on 2 mL of intramuscular Rigivir for two days, followed by an additional 1.5 mL intramuscularly and 0.5 mL intranasally on the third day. She then received weekly doses of Rigivir for the next five months, following a dosing schedule of two weeks of 2 mL intramuscular injection followed by one week of combined 1.5 mL intramuscular/0.5 mL intranasal administration. She then began taking the drug every two weeks, and after another two years, this interval was increased to every three weeks. During a nearly 4-year follow-up, the patient exhibited neither disease progression nor new brain metastases while on chronic Rigivir.

In the first case, OV appears to have limited efficacy on extracranial disease while intracranial metastases appeared to remain stable until after the patient was taken off OV therapy. These findings align with the observation that brain metastases and extracranial metastases have unique molecular profiles [23] and warrant the exploration of OV with combination therapies to better achieve total body remission. The second case is the first of its kind to employ chronic OV after subtotal brain metastasis resection, prolonging survival well above that reported in the literature. Based on these case reports and other published trials, OV appears to be well tolerated with minimal systemic toxicity. Further study in more extensive trials is necessary to better characterize the efficacy of OV against metastatic brain disease from melanoma.

## 6. Overcoming Barriers to Virotherapy

Given the limited available evidence, we must continue to draw upon preclinical studies in animal models as well as extrapolate data from other brain tumor types that may be applied in patients with melanoma brain metastases. The primary challenge of using OV to treat metastatic disease is systemic delivery to specific target organs [50].

There are several obstacles to systemically administered oncolytic viruses. These include complement and antibody-mediated neutralization, as well as uptake by phagocytic macrophages; additionally, further filtering of oncolytic viruses occurs in the liver, spleen, and other tissues, effectively reducing the viral load reaching the target tumor [27]. The BBB serves as an additional obstacle to systemically injected viruses. The question remains whether viruses permeate through the BBB or, perhaps more plausibly, if viruses invade infected endothelium in tumor vasculature before spreading to tumor cells. Nonetheless, viral penetrance into the CNS has been reported with several viruses due to their intrinsic neurovirulent properties, including Semliki Forest virus, vaccinia virus, chimeric vesicular stomatitis virus, parvovirus H-1, Mengovirus, and Seneca Valley virus-001 in both animal models and human trials. There also exists the option of loading oncolytic viruses onto carrier stem cells known as “trojan horses”, which is being investigated for adenovirus strains in two clinical trials involving malignant glioma patients. The authors believe intratumoral and peritumoral resection cavity injections to be the simplest route of oncolytic virus administration for ensuring delivery to tumor cells. In fact, this is the most studied route of administration that appears to be well tolerated in trials exploring OV for the treatment of HGG. To date, 51 clinical trials have been completed investigating OV for HGG treatment [39], and therefore similar investigations modeling these trials should be undertaken for metastatic brain melanoma.

In a preclinical study, Du et al. attempted to improve OV delivery by systemic and intracarotid injection of mesenchymal stem cells (MSCs) infected with oncolytic HSV (oHSV) into mice with melanoma brain metastases [79]. They did not inject MSC-oHSV locally into brain lesions. Mice treated with MSC-oHSV experienced significant remission of their brain lesions compared to mice injected with either purified virus or uninfected MSCs. This finding was consistent among mice with both wildtype *BRAF* and *BRAF* V600E mutations. MSC-oHSV in combination with PD-L1 immunotherapy demonstrated even greater efficacy, with more mice achieving disease remission. Here, immunotherapy may work synergistically with virotherapy, enhancing the immune response to infected tumor cells. Further work exploring intra-arterial delivery of oncolytic viruses is ongoing [80].

Another issue related to OV involves the virus’s ability to disseminate into tumor tissue after reaching the target site. Poor viral penetration is believed to be due to inefficient passage through dense networks of extracellular matrices. To combat this, transgenic viral lines containing structural proteins such as decorin, which can bind collagen fibrils to other matrix components, have been explored. Once attached, decorin hinders extracellular matrix remodeling and allows improved penetration of the oncolytic virus into tumor cells [58]. Given their relative ease of genetic modification, oncolytic viruses have the potential to be enhanced by the insertion of highly specific vectors that serve to achieve a particular goal, whether this goal is optimizing virus delivery or reducing local expression of an oncogenic tumor factor [81,82].

## 7. Future Directions

The field of OV remains in its nascency and is an active area of research. As such, many new oncolytic viruses are being explored in addition to those mentioned in this article. Rigivir should be an area of further clinical exploration the United States, given its preliminary findings overseas. The significant advantage of OV lies in its modifiability, as transgenic variations are developed with relative ease. This enables oncolytic viruses to be genetically engineered with exceptionally high specificity and tumor penetrability, all while avoiding the systemic toxicities inherent in more non-specific cancer therapy options in use today.

The body of research presented thus far on OV indicates that it may be a promising treatment option for metastatic brain melanoma. While the lack of robust data precludes us from formally recommending virotherapy, we believe it to be a promising area of further exploration. Unfortunately, patients with metastatic brain melanoma are often excluded from clinical trials. This contributes to the relative lack of published studies in this patient population and remains one of the most considerable obstacles to our understanding of how best to treat patients with melanoma brain metastases. However, this should not be discouraging, as preclinical studies continue to push new boundaries in introducing either new or genetically enhanced oncolytic viruses. Given the evidence of OV’s efficacy in various other primary and metastatic brain tumors, as well as the promising preliminary findings presented in this review, the authors believe that further exploration of OV for brain metastases of melanoma is warranted.

## 8. Conclusions

OV is the most recent addition to the arsenal of treatments for metastatic melanoma, having already gained FDA approval to treat extracranial metastases. OV is a promising treatment option, as it can be used to treat both *BRAF* and non-*BRAF*-mutated melanomas. Despite the lack of data at this time, OV is ideally suited for metastatic brain disease, given that it is amenable to passage through the BBB based on previous preclinical and clinical reports. In addition, its efficacy in other brain tumor types, most notably high-grade glioma, warrants further exploration of its efficacy in the setting of metastatic brain melanoma. As current data regarding its effectiveness are limited, OV may be employed in specific circumstances in which melanoma patients with metastatic brain disease fail or do not qualify for traditional treatment options.
